# Effect of Carbon Black Nanoparticle on Neonatal Lymphoid Tissues Depending on the Gestational Period of Exposure in Mice

**DOI:** 10.3389/ftox.2021.700392

**Published:** 2021-08-11

**Authors:** Atsuto Onoda, Saki Okamoto, Ryuhei Shimizu, Yasser S. El-Sayed, Shiho Watanabe, Shuhei Ogawa, Ryo Abe, Masao Kamimura, Kohei Soga, Ken Tachibana, Ken Takeda, Masakazu Umezawa

**Affiliations:** ^1^ The Center for Environmental Health Science for the Next Generation, Research Institute for Science and Technology, Tokyo University of Science, Noda, Japan; ^2^ Faculty of Pharmaceutical Sciences, Tokyo University of Science, Noda, Japan; ^3^ Faculty of Pharmaceutical Sciences, Sanyo-Onoda City University, Sanyoonoda, Japan; ^4^ Faculty of Veterinary Medicine, Damanhour University, Damanhour, Egypt; ^5^ Research Institute for Biomedical Sciences, Tokyo University of Science, Noda, Japan; ^6^ Advanced Comprehensive Research Center, Teikyo University, Hachioji, Japan; ^7^ Department of Materials Science and Technology, Faculty of Advanced Engineering, Tokyo University of Science, Katsushika, Japan

**Keywords:** carbon black nanoparticles, air pollution, nanomaterial, neonates, lymphoid tissue development, immune response, non-B/non-T cell, CD3-/B220- phenotype

## Abstract

**Introduction:** Particulate air pollution, containing nanoparticles, enhances the risk of pediatric allergic diseases that is potentially associated with disruption of neonatal immune system. Previous studies have revealed that maternal exposure to carbon black nanoparticles (CB-NP) disturbs the development of the lymphoid tissues in newborns. Interestingly, the CB-NP-induced immune profiles were observed to be different depending on the gestational period of exposure. It is important to identify the critical exposure period to prevent toxic effects of nanoparticles on the development of the immune system. Therefore, the present study was aimed to investigate the effect of CB-NP on the development of neonatal lymphoid tissues in mice, depending on the gestational period of exposure.

**Methods:** Pregnant ICR mice were treated with a suspension of CB-NP (95 μg/kg body weight) by intranasal instillation; the suspension was administered twice during each gestational period as follows: the pre-implantation period (gestational days 4 and 5), organogenesis period (gestational days 8 and 9), and fetal developmental period (gestational days 15 and 16). The spleen and thymus were collected from offspring mice at 1, 3, and 5-days post-partum. Splenocyte and thymocyte phenotypes were examined by flow cytometry. Gene expression in the spleen was examined by quantitative reverse transcription-polymerase chain reaction.

**Results:** The numbers of total splenocytes and splenic CD3^−^B220^−^ phenotype (non-T/non-B lymphocytes) in offspring on postnatal day 5 were significantly increased after exposure to CB-NP during the organogenesis period compared with other gestational periods of exposure and control (no exposure). In contrast, expression levels of mRNA associated with chemotaxis and differentiation of immune cells in the spleen were not affected by CB-NP exposure during any gestational period.

**Conclusion:** The organogenesis period was the most susceptible period to CB-NP exposure with respect to lymphoid tissue development. Moreover, the findings of the present and previous studies suggested that long-term exposure to CB-NP across multiple gestational periods including the organogenesis period, rather than acute exposure only organogenesis period, may more severely affect the development of the immune system.

## Introduction

Child health promotion is an important community goal to realize a sustainable society for future generations. The increasing prevalence of allergic diseases such as asthma, eczema, and hay fever is a serious problem (Devereux, 2006), and implicates disruptions in the immune system. Since the immune system of newborns is immature and susceptible to exogenous factors, perinatal exposure to endocrine disruptors (Camacho et al., 2004; Mustafa et al., 2009), chemical substances (Midoro-Horiuti et al., 2010), and heavy metals (Jedrychowski et al., 2015) interferes with the neonatal development of lymphoid tissues. In addition to these factors, emerging research has suggested that air pollution increases the risk of developing allergic diseases in the childhood (Kim et al., 2018; Amazouz et al., 2021).

Among air pollutants, suspended particles, including fine particle matter (PM_2.5_), are a major public health concern. The toxicological properties of particles change as they approach the nanometer size range. Inhaled nanoparticles can reach the alveolar region, which is the deepest area of the respiratory organ (Oberdörster et al., 2002), translocate into the bloodstream (Choi et al., 2010), and circulate throughout the body (Kreyling et al., 2002). An *ex vivo* study using the human placenta demonstrated that nanoparticles, less than 240 nm, can pass through the blood-placental barrier (Wick et al., 2010). Moreover, animal studies have shown that nanoparticle injected during the fetal period was detected in the fetus (Takeda et al., 2009; Yamashita et al., 2011) and perinatal exposure to nanoparticles induced abnormal development of fetal organs, including the central nervous system (Onoda et al., 2020), genital organs (Takeda et al., 2009; Kubo-Irie et al., 2014), and the liver (Jackson et al., 2013). In addition to these organs, nanoparticles invading fetus may affect the fetal immune system and cause allergic diseases in the childhood owing to the strong link between the exposure to nanoparticles and immune reactions (Zolnik et al., 2010) and inflammatory responses (Sun et al., 2013). Moreover, maternal exposure to low doses of air particulate matter has been identified as a cause of an increase in susceptibility to the allergic diseases of airways in the offspring (Fedulov et al., 2008). Findings of a cohort study have suggested that exposure to particulate air pollution during pregnancy may induce neonatal airway inflammation associated with allergic diseases in the childhood (Latzin et al., 2009). Therefore, it is important to reveal the mechanisms of immunotoxicity caused by nanoparticles and the effects of maternal exposure to them.

To evaluate the effects of nanoparticles on the development of the immune system, we have investigated the effects of maternal exposure to carbon black nanoparticles (CB-NP), model particles of air pollution (Long et al., 2013), on the development of various organs related to immune responses of the offspring. Upon exposure of 11-weeks old pregnant ICR mice to CB-NP (95 μg/kg body weight) during early- and middle-gestation period (gestational days 5 and 9, respectively), a decrease in the number of CD3^+^, CD4^+^, and CD8^+^ T cells in the spleen was observed in infantile mice (Shimizu et al., 2014). In contrast, maternal exposure to the same dose of CB-NP during middle- and late-gestation period (gestational days 9 and 15, respectively) induced an increase in the number of total thymocytes, including CD4^−^CD8^−^ and CD4^+^CD8^+^ cells, and splenic lymphocytes, including CD4^−^CD8^−^, CD3^+^, B220^+^, and CD3^−^ B220^−^ cells, in infantile mice, suggestive of stimulation of immature splenocytes (El-Sayed et al., 2015). Interestingly, the findings of the studies indicated that immune responses elicited by CB-NP might be dependent on the stage of gestation to which they were exposed. Therefore, this critical exposure period is important to understand the mechanisms underlying the toxic effects of nanoparticles on the development of the immune system. Here, we reported differential effects of CB-NP exposure during each gestation period on the thymus, the central tissue of the immune system, and the spleen, the peripheral tissue of the immune system, in infantile mice. The exposure period was divided into three periods: pre-implantation period, organogenesis period, and fetal developmental period.

## Materials and Methods

### Preparation of Carbon Black Nanoparticles Suspension

The CB-NP suspension was prepared according to previously reported methods (Onoda et al., 2014; Shimizu et al., 2014; El-Sayed et al., 2015). Printex 90 (CB-NP; primary particle diameter of approximately 14 nm and surface area of 295–338 m^2^/g) was obtained from Degussa Ltd. (Frankfurt, Germany). Constituent elements of CB-NP are >99% carbon, 0.82 weight percent (wt%) nitrogen, 0.01wt% hydrogen, and <1wt% organic and inorganic impurities. Before intranasal instillation, CB-NP were suspended at a concentration of 5 mg/ml in ultrapure water, sonicated for 30 min using an ultrasonicator, and immediately filtered through a 450 nm filter (S-2504, Kurabo Co., Ltd., Osaka, Japan) to remove agglomerated particles, as previously described (Shimizu et al., 2014; El-Sayed et al., 2015).

As previously described (Shimizu et al., 2014), the distribution of hydrodynamic diameter of CB-NP in the suspension was measured by dynamic light scattering (NANO-ZS, Sysmex Co., Kobe, Hyogo, Japan) using the Rayleigh-Debye equation, and the estimated mode value was 68 nm. Similarly, field-emission scanning electron microscopy (FE-SEM, JSM-6500F, JEOL Ltd., Tokyo, Japan) on a silicon wafer showed small agglomerates having a characteristic diameter of approximately 50–500 nm (Shimizu et al., 2014). The primary and secondary diameters of CB-NP were smaller than the ones of inorganic nanoparticles used in the previous study that revealed the placental translocation of nanoparticle (Wick et al., 2010). CB-NP concentration in the suspension was calculated as 95 μg/ml by the peak area of the carbon signal (2.77 keV) obtained using an FE-SEM (JSM-6500F) with an attached energy-dispersive X-ray analyzer (Onoda et al., 2014).

### Animals and Treatments

Thirty-one pregnant ICR mice at 11 weeks of age were purchased from Japan SLC Inc. (Shizuoka, Japan) and were randomly divided into control group (C group; *n*=8), pre-implantation period exposure group (P group; *n*=9), organogenesis period exposure group (O group; *n*=7), and fetal developmental period exposure group (F group; *n*=7). The mice were housed in a room at a controlled temperature (22–24°C) and humidity (50–60%), with a 12-h dark/light cycle, and were given ad libitum access to food and water.

**FIGURE 1:**
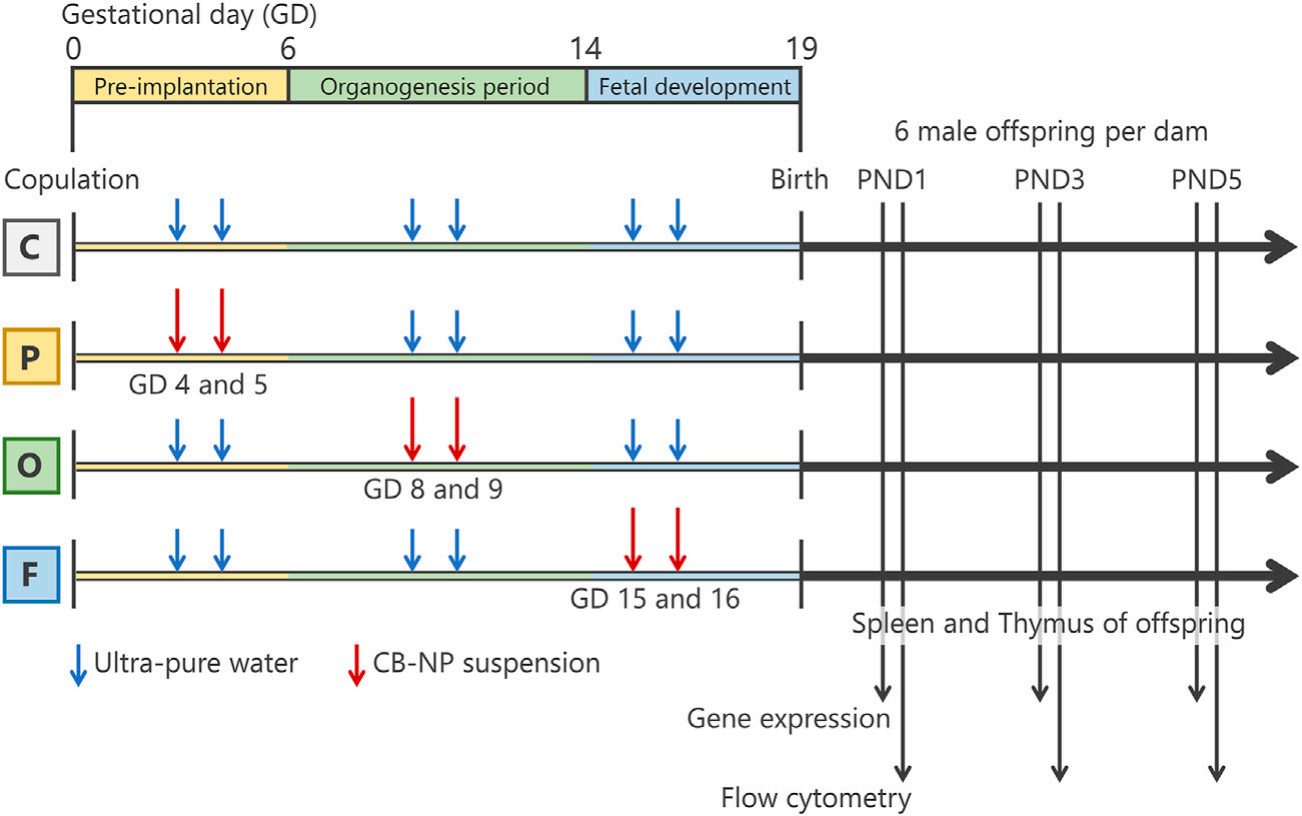
Summarized scheme of animal treatments and sample collection. Pregnant mice were randomly divided into four groups; control group (C group; *n*=8), pre-implantation period exposure group (P group; *n*=9), organogenesis period exposure group (O group; *n*=7), and fetal developmental period exposure group (F group; *n*=7). The pregnant mice were intranasally exposed to carbon black nanoparticle suspension (95 μg/kg body weight) at gestational days 4 and 5 for the P group, gestational days 8 and 9 for the O group, and gestational days (GDs) 15 and 16 for the F group. The control group were treated with the same volume of ultrapure water each time. After childbearing, six male offspring per 1 dam were randomly selected and their spleen and thymus were collected at postnatal day (PND) 1, 3, and 5 for flow cytometry and gene expression analysis.

The amount and method of exposure of pregnant mice to CB-NP were the same as described in previous studies (El-Sayed et al., 2015; Shimizu et al., 2014), except for gestational periods of exposure. Before exposure, the pregnant mice were placed in an anesthesia box filled with halothane and removed from the box after they began to sleep. Immediately, the sleeping mice were laid on their backs and exposed to the CB-NP suspension (1 ml/kg body weight) by intranasal instillation through both nostrils. Intranasal instillation was performed at gestational days 4 and 5 for the P group, gestational days 8 and 9 for the O group, and gestational days 15 and 16 for the F group (Figure 1). The total dose of CB-NP was 190 μg/kg body weight per pregnant mouse. Control mice were treated with the same volume of ultrapure water each time using the same exposure method. After childbearing, six male offspring per 1 dam were randomly selected and their thymus and spleen were collected at postnatal day (PND) 1, 3, and 5 under anesthesia with sodium pentobarbital for flow cytometry and gene expression analysis. Each offspring mouse was used for one analysis. The differential effects of CB-NP exposure depending on the gestational period of exposure was comparatively investigated by observing the lymphatic cell phenotype of the thymus and the spleen of offspring mice at 1, 3, and 5 days of age, which is useful for screening of developmental immunotoxicity following exposure to inorganic nanoparticles as shown by previous studies (Shimizu et al., 2014; El-Sayed et al., 2015).

All animal experiments were treated and handled in accordance with the Animal Research: Reporting *In Vivo* Experiments (ARRIVE) guidelines for the care and use of laboratory animals (Kilkenny et al., 2011), and with the approval of the Institutional Animal Care and Use Committee of Tokyo University of Science. All efforts were made to minimize the number of mice used and the suffering experienced by them.

### Near-Infrared Imaging of Instilled Nanoparticles in the Airways of Mice

To investigate the distribution of intranasally instilled inorganic nanoparticles into the lung tissues, adult mice were treated with intranasal instillation of an aqueous suspension of NaYF4 co-doped with Yb^3+^ and Er^3+^, which emits near-infrared luminescence at 1,550 nm by irradiation with 980 nm light, as a model of inorganic nanoparticles (120 nm, 10 mg/ml) (Kamimura et al., 2017). The distribution of the instilled nanoparticles was observed using a near-infrared camera (Xenics, Leuven, Belgium) under irradiation with near-infrared light of a wavelength of 976 nm.

### Hematoxylin and Eosin Staining of the Nasal Cavity of Dams

The nasal cavity of the dams collected after 10-days of final instillation (5-days after birth of the offspring) was fixed in the 0.1 mol/L phosphate buffer (pH 7.4) containing 4% paraformaldehyde for 24 h. The fixed tissues were decalcified using 15% formic acid in the 10% formaldehyde for 48 h. After softening the bone tissues, the nasal cavity was divided into three regions (nasal vestibule, respiratory region, and olfactory region). The tissues were embedded into paraffin after dehydration using ethanol and xylene. The tissues cut into 4 μm sections by microtome (TTM-200, Sakura Finetek Japan Co., Ltd., Tokyo, Japan) and stained with hematoxylin and eosin (H&E) for histopathological analysis.

### Flow Cytometry

Fluorescein isothiocyanate (FITC)-conjugated anti-CD3 (2C11) and anti-CD4 (GK1.5) antibodies purified from hybridoma culture supernatants were provided by the Division of Immunobiology, Research Institute for Biological Sciences, Tokyo University of Science (Chiba, Japan) (Watanabe et al., 2012). Phycoerythrin (PE)-conjugated anti-B220 (RA3-6B2) and anti-CD8 (53–6.7) antibodies were purchased from BD Bioscience Co. (San Jose, CA, United States). Cells of the thymus and spleen collected from individual male offspring at PND 1, 3, and 5 mice were dispersed in a single cell using frosted glass slides and suspended in RPMI-1640 medium at a concentration of 1 × 106 cells/ml. The suspensions were washed with fluorescence-activated cell sorting (FACS) medium (phosphate-buffered saline containing 1% fetal bovine serum and 0.1% sodium azide), treated with anti-FcR (2.4G2) to block non-specific binding (Watanabe et al., 2012), and then stained with fluorescent-conjugated antibodies. The cells were then washed, resuspended in the medium, and prepared for flow cytometric analysis.Fluorescence data of 10,000 lymphocyte events per sample were acquired with BD FACSCantoTM II (BD Biosciences, San Jose, CA, United States) and analyzed using FlowJo 7.2.2.2. software (Tomy Digital Biology Co., Ltd., Tokyo, Japan). The lymphocyte subpopulation was discriminated from other cells, including monocytes and granulocytes, using peak area of forward- and side-scatter signal (FSC-A and SSC-A). Also, dead cells were excluded using FSC-A gating and propidium iodide staining. In the lymphocyte subpopulation, the numbers of CD4^−^CD8^−^, CD4^+^CD8^−^, CD4^−^CD8^+^ cells, and CD4^+^/CD8^+^ ratio in the thymus and CD3^−^B220^−^, CD3^+^B220^−^, CD3^−^B220^+^, CD4^−^CD8^−^, CD4^+^CD8^−^, and CD4^−^CD8^+^ cells in the spleen were calculated based on the percentage of each subpopulation.

### Total RNA Extraction and Quantitative Reverse Transcription-Polymerase Chain Reaction

Spleen tissues were homogenized in Isogen II to extract total RNA (Nippon Gene Co., Ltd., Tokyo, Japan) according to the manufacturer’s protocol, and suspended in RNase-free water. RNA quantification was performed by spectrophotometry at OD260 in a BioPhotometer plus (Eppendorf, Hamburg, Germany). RNA extracted from each sample was used for qRT-PCR analyses.

Total RNA (1 μg) from each sample was reverse-transcribed with M-MLV reverse transcriptase (Invitrogen Co., Carlsbad, CA, United States) to generate complementary DNA according to the manufacturer’s instructions. qRT-PCR was performed in duplicate using SYBR Green Real-Time PCR Master Mix (Toyobo Co. Ltd. Osaka, Japan) and primers (Fasmac Co., Ltd. Kanagawa, Japan) for the indicated genes (Table 1). In the present study, we chose genes associated with major chemokines for recruitment of lymphocyte subsets and master regulators for differentiation of each lymphocyte. The target gene expression levels were normalized to the expression level of the housekeeping gene, glyceraldehyde 3-phosphate dehydrogenase *(Gapdh)*.

**TABLE 1:** Primer and probe sequences for quantitative reverse transcription-polymerase chain reaction analyses.

Gene	Accession No		Sequence (5' > 3′)	Tm (°C)
*IL7*	NM_008371.4	F:	ACCTCCCGCAGACCATGT	58
		R:	CAG​AAC​AAG​GAT​CAG​TGG​AGG​A	
*IL15*	NM_001254747.1	F:	ACC​AGC​CTA​CAG​GAG​GCC​AAG​AAG	62
		R:	TGA​GCT​GGC​TAT​GGC​GAT​GGG	
*GATA3*	NM_008091.3	F:	CAA​CCT​TTT​GGC​TGC​ACC​CCA	62
		R:	CAT​ACC​TGG​CTC​CCG​TGG​TGG​G	
*GAPDH*	NM_008084.2	F:	AGC​CCT​GGG​AGT​TCC​TGG​TCG​G	60
		R:	GGA​TGC​ATT​GCT​GAC​AAT​CT	
*CCR7*	NM_008084.2	F:	GCA​CCA​TGG​ACC​CAG​GGA​AAC​C	60
		R:	GTC​CAC​CGT​GGT​ATT​CTC​GCC​G	
*CCL19*	NM_008084.2	F:	CTG​CCA​AGA​ACA​AAG​GCA​ACA​GCA​C	60
		R:	CAG​AGC​ATC​AGG​AGG​CCT​GGT​C	

### Statistical Analysis

All data are presented as mean ± standard deviation (SD), and the levels of significance are cited. R version 3.6.3 (https://www.r-project.org/) was used for statistical analyses. Significant effects and interaction of gestational periods of CB-NP exposure and age on number and sex ratio of newborns per dam, body weight of offspring, and flow cytometry data, and mRNA expression levels were identified by two-way repeated-measures analysis of variance (ANOVA). The ANOVA was combined with the Tukey-Kramer post-hoc test when appropriate. The significance level was set at *p* < 0.05.

## Results

### Translocation of Nanoparticle to the Lung

**FIGURE 2:**
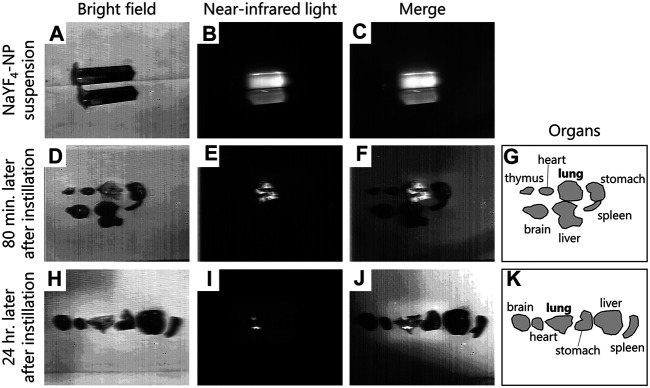
Distribution of intranasally instilled nanoparticle throughout the lung in mice. **(A–C)**: Images of NaYF_4_: Yb^3+^, Er^3+^ nanoparticle dispersions and their luminescence under irradiation with near-infrared light (976 nm). **(D–G)**: Distribution of nanoparticles in each organ after 80 min of intranasal instillation of the suspension. **(H–K)**: Distribution of nanoparticles in each organ after 24 h of intranasal instillation of the suspension. **(A,D,E)**: Pictures of nanoparticles dispersions and the organs captured under bright field. **(B,E,I)**: Luminescence under irradiation with near-infrared light. **(C,F,J)**: Merge images of A and B, D and E, and H and I, respectively. **(G,K)**: Illustration showing the positions of the organs in the D and H pictures. Fluorescence images were obtained using a near-infrared camera with an InGaAs sensor (integration time: 500 ms), scanned under irradiation with near-infrared light (976 nm, 4.2 W) with a Galvano mirror.

The distribution of nanoparticles instilled into the nasal cavity of mice was investigated using an aqueous suspension of fluorescent-labelled NaYF4, model inorganic nanoparticles, that fluoresces in over-1000 nm near-infrared region. Upon irradiation of the nanoparticle suspension with near-infrared light (976 nm), which highly penetrates biological tissues, the emission of infrared fluorescence peaked at 1,550 nm (Figures 2A–C). The fluorescence derived from the nanoparticles was observed throughout the lung at 80 min after intranasal instillation, while no fluorescence was observed in other organs (Figures 2D–G). In addition, fluorescence remained in the lungs at 24 h after instillation (Figures 2H–K). The images have shown that the intranasal instillation can transport the nanoparticle to the respiratory organ but not the gastric organ and the translocated nanoparticle to the respiratory organ was gradually removed from the lung over 24 h. Since this ex-vivo imaging analysis using near-infrared light can capture at only so far nanoparticle-accumulated sites, it was not possible to evaluate the translocation of nanoparticle to the extrapulmonary organs, including the placenta, even if a small amount of the nanoparticle reached the organs.

### Histopathology of the Nasal Cavity of Dams

**FIGURE 3:**
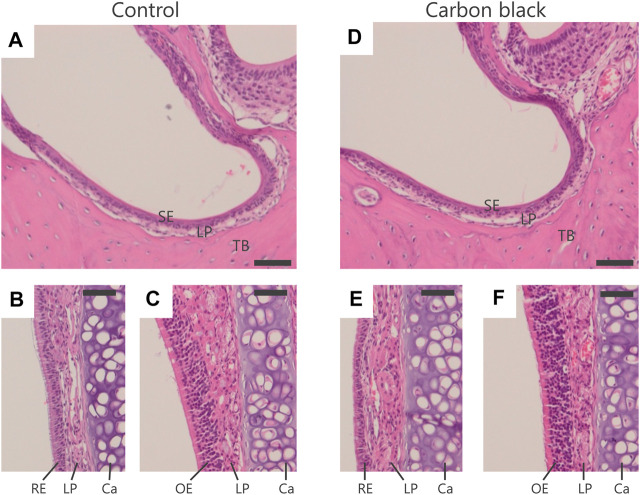
Histological analysis of nasal cavity of mother mice. **(A–C)**: Control group. **(D–F)**: CB-NP-exposed group. **(A,D)**: Nasal vestibule region. **(B,E)**: Respiratory region. **(C,F)**: Olfactory region. The scale bars represent 50 mm. Paraffin sections (4 μm) of the nasal cavity of mother mice (*n*=5) were stained with hematoxylin and eosin after 10-days of final instillation. Deposition of CB-NP and histopathological alteration was not detected in the nasal cavity and/or surrounding regions of mother mice. Abbreviations: RE; Respiratory Epithelium, LP; Lamina Propria, Ca; Cartilage, OE; Olfactory Epithelium, SE; Squamous Epithelium, TB; Turbinate Bones.

In mother mice, CB-NP were not deposited in the nasal cavity or surrounding regions (Figure 3). In addition, no inflammation was observed in the tissues (Figure 3). The results suggested that the nasal cavity was not damaged by instillation or recovered during gestation.

### Number of Newborns per Dam, Sex Ratio, and Body Weight

No deaths caused by intranasal instillations of CB-NP in pregnant mice were observed during each exposure period. There were no significant differences in the number of offspring per dam and sex ratio of newborns (Table 2), and offspring body weights at PND 1, 3, and 5 among each group (Table 3).

**TABLE 2:** Number and sex ratio of offspring.

Group	Number of dams	Number of offspring per mother mouse	Sex ratio (%) [male/(male + female) x 100]
Control	11	14.82 ± 2.14	53.37 ± 2.14
Pre-implantation	13	15.08 ± 1.04	55.10 ± 1.04
Organogenesis	13	14.45 ± 1.39	47.87 ± 1.39
Fetal developmental	10	13.60 ± 2.41	50.76 ± 2.41

**TABLE 3:** Body weight [g] of offspring at postnatal days (PND) 1, 3, and 5.

Group	PND 1		PND 3		PND 5	
	Male	Female	Male	Female	Male	Female
Control	1.87 ± 0.18	1.77 ± 0.18	2.56 ± 0.42	2.47 ± 0.41	4.10 ± 0.75	3.97 ± 0.82
Pre-implantation	1.92 ± 0.13	1.79 ± 0.11	2.57 ± 0.23	2.65 ± 0.31	4.20 ± 0.35	4.11 ± 0.41
Organogenesis	1.84 ± 0.18	1.87 ± 0.23	2.80 ± 0.33	2.77 ± 0.30	4.06 ± 0.70	4.08 ± 0.52
Fetal developmental	1.89 ± 0.16	1.89 ± 0.13	2.75 ± 0.42	2.66 ± 0.36	4.18 ± 0.65	4.17 ± 0.53

### Number of Total Lymphocytes and Each Immunophenotype in the Thymus and Spleen

To identify the critical gestational periods, we evaluated the total number and immunophenotyping of lymphocytes in the thymus and spleen of offspring.

**FIGURE 4:**
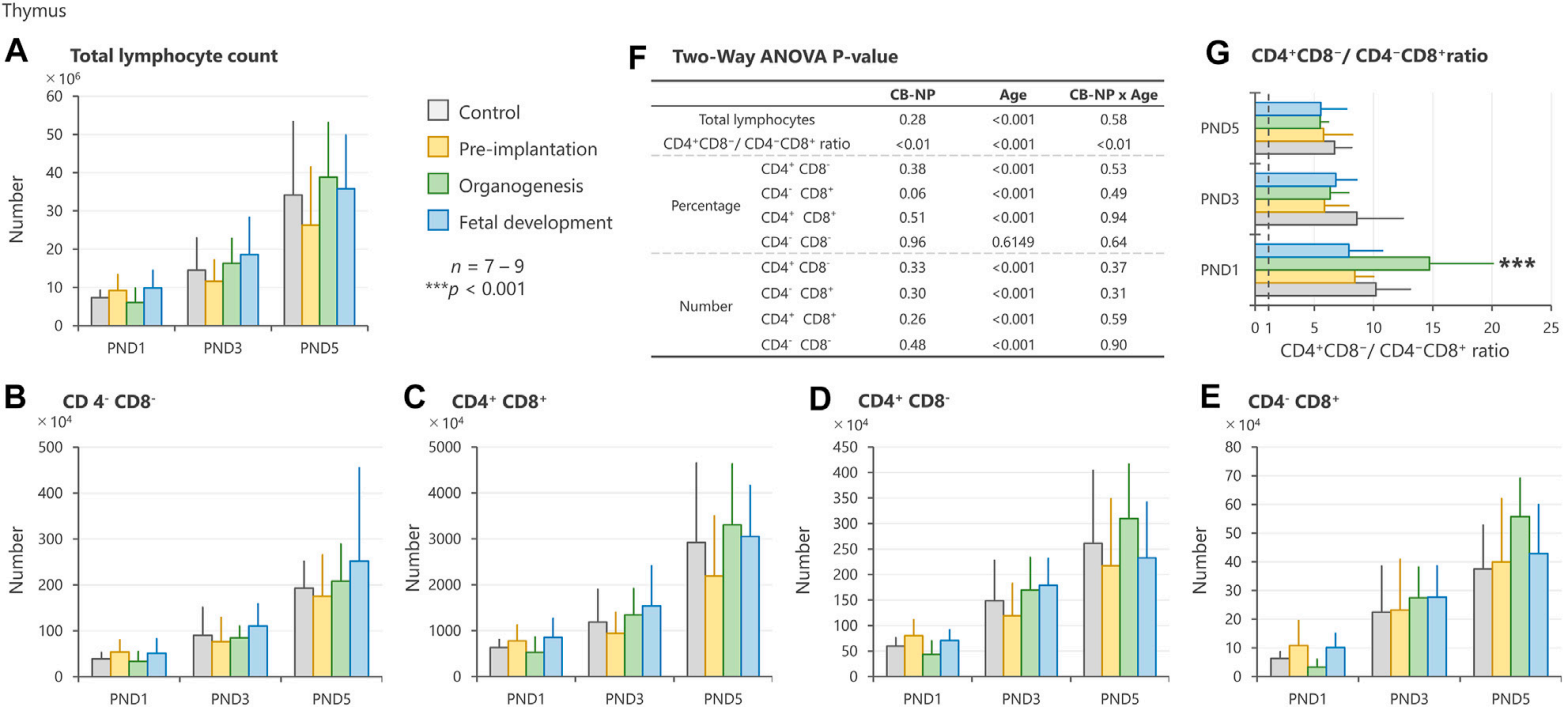
Effect of maternal exposure to carbon black nanoparticle (CB-NP) on the number and percentage of each lymphocyte in the thymus of offspring at postnatal day 1, 3 and 5, as measured by flow cytometry. **(A)**: Number of total lymphocytes in the thymus. **(B**–**E)**: Number of each lymphocyte calculated based on the total lymphocytes and percentage of each cell type. **(F)**: All *p-values* calculated by two-way ANOVA. Two-way ANOVA showed no significant effect of CB-NP exposure on the number of **(A)** total lymphocytes [F (3, 79) = 1.32; *p* = 0.28], **(B)** CD4^−^CD8^−^ [F (3, 79) = 0.83; *p* = 0.48], **(C)** CD4^+^CD8^+^ [F (3, 79) = 1.38; *p* = 0.26], **(D)** CD4^+^CD8^−^ [F (3, 79) = 0.82; *p* = 0.33], and **(E)** CD4^−^CD8^+^ [F (3, 79) = 1.22; *p* = 0.30]. **(F)** A significant effect of CB-NP exposure was detected on the CD4^+^CD8^−^/CD4^−^CD8^+^ ratio [F (3, 79) = 4.12; **p = 0.0090] with significant exposure/age interaction [F (6, 79) = 3.78; **p = 0.0023]. The Tukey post-hoc test showed that **(G)** the CD4^+^CD8^−^/CD4^−^CD8^+^ ratio after CB-NP exposure during the organogenesis period was higher than that of the control (**p = 0.0081), pre-implantation period (***p < 0.001), and fetal developmental period groups (***p < 0.001) on postnatal day 1. Values are expressed as mean ± SD.

In the thymus, no significant changes were detected in the number of total lymphocytes and specific phenotype (Figures 4A–E). However, exposure to CB-NP during the organogenesis period induced a high ratio of CD4^+^CD8^−^/CD4^−^CD8^+^ in offspring at PND 1 compared with other groups (Figures 4F,G).

**FIGURE 5:**
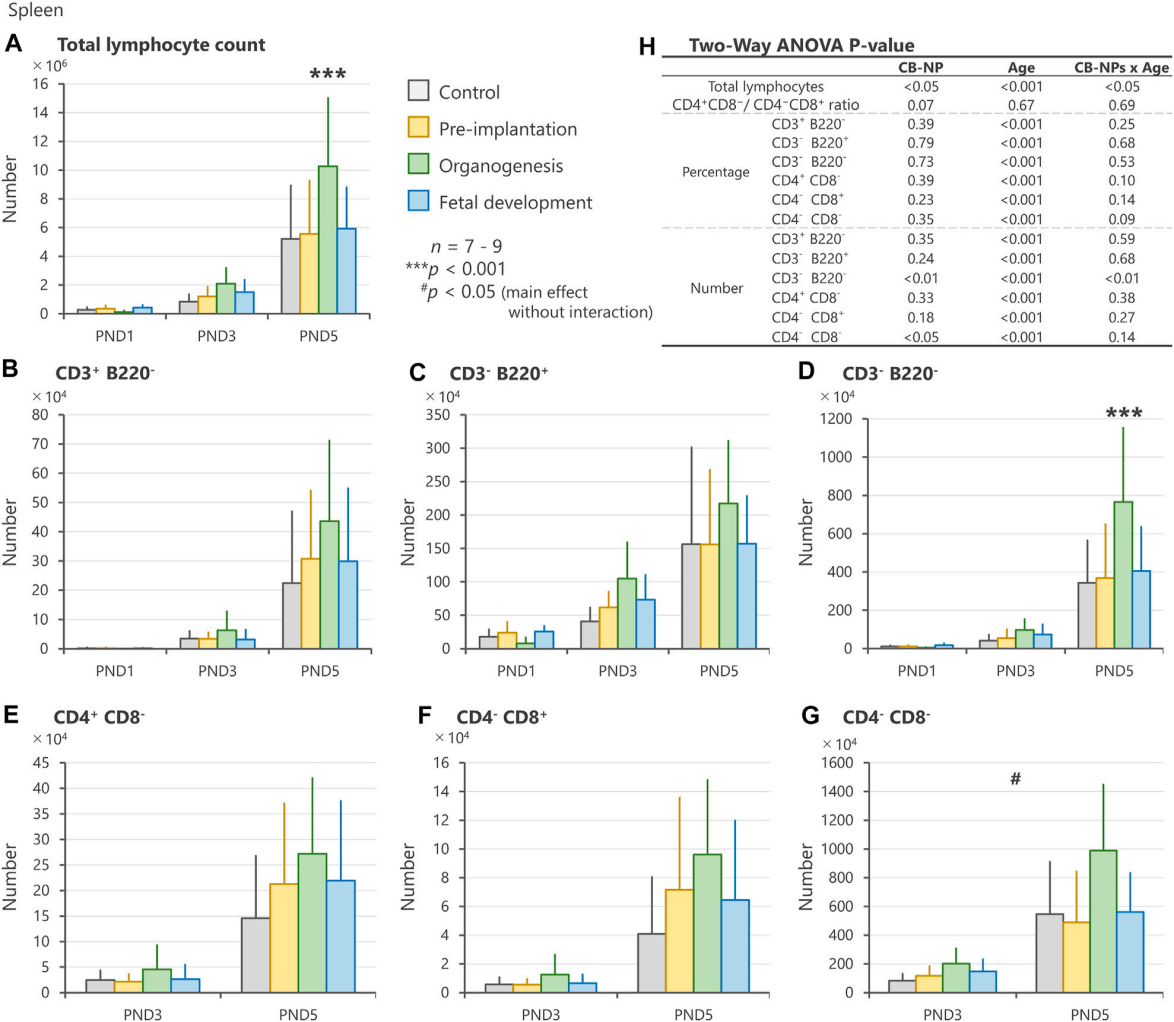
Effect of maternal exposure to carbon black nanoparticle (CB-NP) on the number and percentage of each lymphocyte in the spleen of offspring at postnatal day 1, 3 and 5, as measured by flow cytometry. **(A)**: Number of total lymphocytes in the spleen. **(B**–**G)**: Number of each lymphocyte calculated based on the total lymphocytes and percentage of each cell type. **(H)**: All *p-values* calculated by two-way ANOVA. Two-way ANOVA showed significant effects of CB-NP exposure on the number of **(A,H)** total lymphocytes [F (3, 79) = 3.66; *p = 0.016] with exposure/age interaction [F (6, 79) = 2.53; *p = 0.027]; **(D,H)** CD3^−^B220^−^ [F (3, 79) = 4.39; **p = 0.0066] with significant exposure/age interaction [F (6, 79) = 5.12; **p = 0.0058]; and **(G,H)** CD4^−^CD8^−^ [F (3, 52) = 3.94; #*p* = 0.013] without exposure/age interaction [F (6, 52) = 1.89; *p* = 0.14] in the spleen, and no significant effect of the exposure on the number of **(B)** CD3^+^B220^−^ [F (3, 79) = 1.11; *p* = 0.35]; **(C)** CD3^−^B220^+^ [F (3, 79) = 1.44; *p* = 0.24]; **(E)** CD4^+^ CD8^−^ [F (3, 52) = 1.18; *p* = 0.33]; and **(F)** CD4^−^CD8^+^ [F (3, 52) = 1.68; *p* = 0.18]. The Tukey post-hoc test showed that the number of **(A)** total lymphocytes in the organogenesis period group was significantly increased compared with the control (***p < 0.001), pre-implantation period (***p < 0.001), and fetal developmental period groups (**p = 0.0057) on postnatal day 5. In addition, the Tukey post-hoc test indicated that the number of **(D)** CD3^−^B220^−^ cells in the organogenesis period group was significantly increased compared with the control (***p < 0.001), pre-implantation period (***p < 0.001), and fetal developmental period groups (**p = 0.0012) on postnatal day 5. Values are expressed as mean ± SD.

In the spleen, CB-NP exposure significantly affected the number of total lymphocytes and CD3^−^B220^−^ phenotype with respect to CB-NP exposure (gestational periods)/age (PND) interaction. The numbers of total lymphocytes and CD3^−^B220^−^ phenotype in the lymphocyte subpopulation were significantly increased on PND 5 after exposure to CB-NP during the organogenesis period compared with other groups (Figures 5A,D). Moreover, the CD4^−^CD8^−^ phenotype in the lymphocyte subpopulation tended to increase after maternal exposure to CB-NP during the organogenesis period (Figures 5G,H). Since CD4^−^CD8^−^ subpopulation includes various types of lymphocytes such as double-negative T cells, thymic-derived immature T cells, B cells and non-T/non-B cells, we analyzed CD3/CD220 lymphocyte populations in the same individuals. The analysis indicated that the increase in the CD4^−^CD8^−^ subpopulation was due to the increase in the CD3^−^B220^−^ lymphocytes (non-T/non-B cells), but not CD3^+^ lymphocytes (T cell) or B220^+^ lymphocytes (B cell). Both lymphoid tissues were significantly affected by the organogenesis exposure to CB-NP. In contrast, neither the total number nor the specific lymphocyte population changed significantly after exposure to CB-NP during the pre-implantation and fetal developmental periods (Figures 5A–H).

### Quantitative Analysis of Splenic mRNA Expression

**FIGURE 6:**
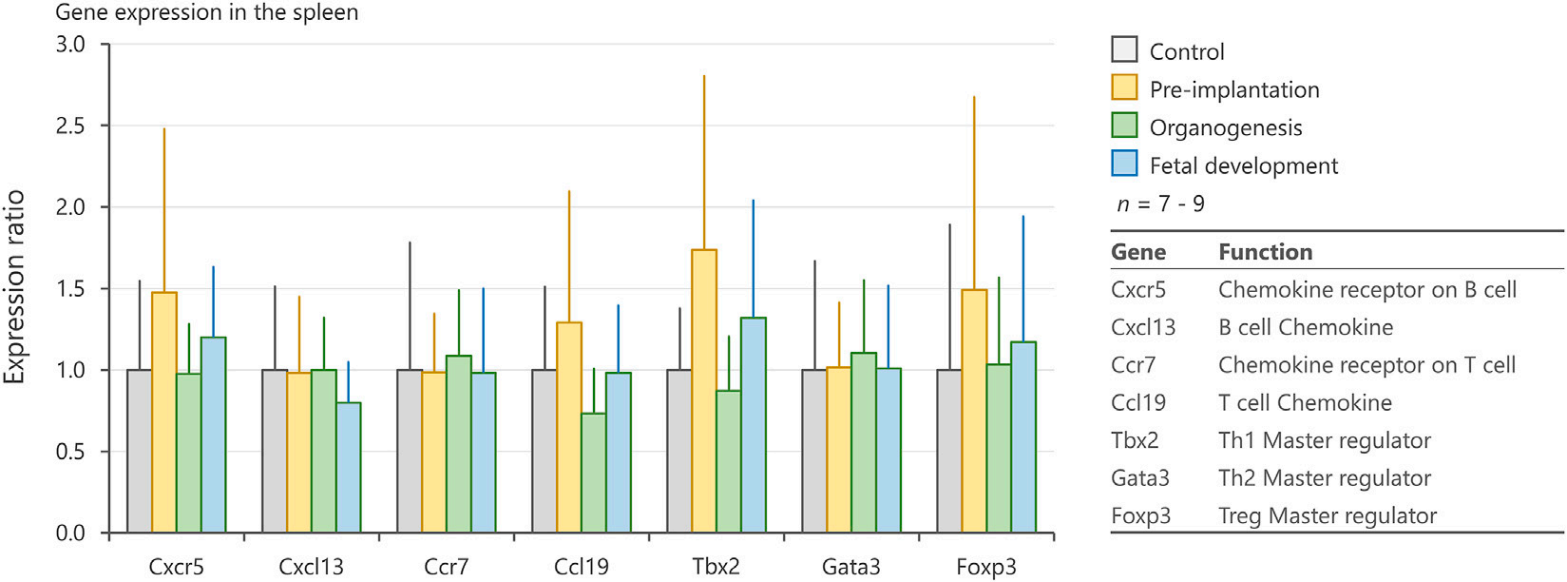
Expression levels of genes related to chemotaxis and differentiation of immune cells in the spleen. mRNA expression levels of *Cxcr5*, *Cxcl13*, *Ccr7*, *Ccl19*, *Tbx2*, *Gata3*, and *Foxp3* in the spleen on postnatal days 1, 3, and 5, as measured by qRT-PCR. Values are expressed as the mean ± SD.

To elucidate the mechanisms underlying the changes in the number of splenic lymphocytes, we evaluated the expression levels of genes associated with cell migration (*Cxcr5*, *Cxcl13*, *Ccr7*, and *Ccl19*) and differentiation (*Tbx2*, *Gata3*, and *Foxp3*) in the spleen. No significant differences were observed among groups with respect to these genes (Figure 6).

## Discussion

**FIGURE 7:**
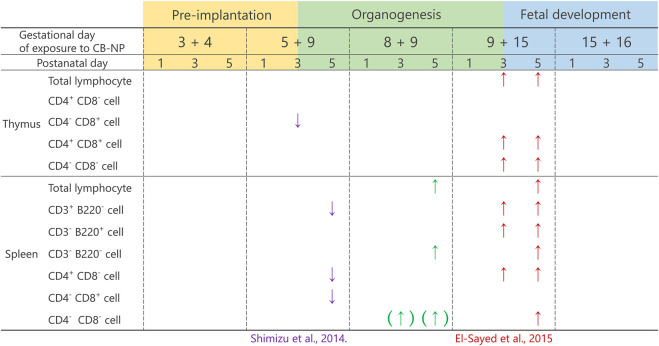
Summary of the effects of maternal exposure to carbon black nanoparticle on lymphoid tissues. The present study and the previous studies have shown that the effects of maternal CB-NP exposure on the population of lymphocytes in the thymus and spleen were different depending on the gestational periods of the exposure. The effects were greater for exposures that include the organogenesis period. The evidence suggests that long-term exposure across multiple gestational periods including the organogenesis period may cause serious effects on the development of immune tissues compared with acute exposure. The arrows indicate a significant increase or decrease in the cell number. The arrows with brackets indicate a tendency of the increase or decrease in the cell number.

Particulate air pollutants, which induce adverse effects on lymphoid tissues, have been recognized as potential risk factors for allergic diseases (Kim et al., 2018; Amazouz et al., 2021). Previous studies using adult animals have shown that nanoparticles approaching pulmonary tissues were translocated to the surrounding lymph nodes and exaggerated inflammatory responses (Shwe et al., 2005; Shimada et al., 2006). Moreover, antigen sensitization after nanoparticle exposure stimulated immune cells more severely than after single exposure to antigens or nanoparticles (van Zijverden et al., 2001; de Haar et al., 2005; Nygaard et al., 2009). Nanoparticle exposure potentially causes adverse effects on the immune system, and therefore, the risk of allergic and infectious diseases might be increased. Thus, studying immune response is crucial to understand the adverse outcomes of nanoparticle exposure. Besides postnatal exposure to nanoparticles, prenatal exposure is likely to cause impairment of the immune system, resulting in the frequent onset of allergic diseases in the childhood (Dietert and Holsapple, 2007). In other words, the immune system may be more susceptible to nanoparticles in the developmental stage than after maturity. In fact, maternal exposure to diesel exhaust and tobacco smoke, containing particulate matter, has been identified as a risk factor for allergic immune responses in offspring (Watanabe and Ohsawa, 2002; Singh et al., 2003; Penn et al., 2007). Investigation of the developmental immunotoxicity induced by maternal exposure to nanoparticles can provide essential information to establish preventive methods against the development of allergic diseases. The present investigation, using CB-NP as a model of particulate air pollutants, demonstrated that the adverse effects of CB-NP on infantile lymphoid tissues were different depending on the gestation period of exposure. The organogenesis period was observed to be the most susceptible period with regard to the lymphocyte population, even at low doses of CB-NP exposure corresponding to the environmental reference value (35 µg/m^3^). In detail, CB-NP exposure on gestational days 8 and 9 during the organogenesis period induced significant increase in the number of lymphocytes, particularly CD3^−^B220^−^ phenotype, in the spleen of offspring (Figure 7), even though their body weight did not change significantly. The organogenesis period (gestational days 6–14 in mice) in humans corresponds to 4–13 weeks of pregnancy, when the mother is often unaware of her pregnancy (O’Rahilly and Muller, 2010; Xue et al., 2013). These findings suggest the importance of focusing on the organogenesis periods for the evaluation and management of developmental immunotoxicity of nanoparticles as well as chemical toxic substances.

While our findings indicated an increase in the number of lymphocytes in the spleen by CB-NP exposure on gestational days 8 and 9, a previous study displayed a decrease in the number of lymphocytes in the spleen and thymus on gestational days 5 and 9 in pregnant mice treated with the same exposure (Shimizu et al., 2014) (Figure 7). Another study demonstrated an increase in the number of nearly all phenotypes of lymphocytes in the spleen and thymus along with dysregulation of the gene expression related to the development of lymphocytes (*IL-7* and *Themis*), by CB-NP exposure on gestational days 9 and 15 in pregnant mice treated with the same exposure (El-Sayed et al., 2015) (Figure 7). On the contrary, the present study showed only a moderate increase in the number of lymphocytes in the spleen without dysregulation of gene expression. The evidence suggests that exposure to nanoparticles across several gestational periods including the organogenesis period may cause different biological effects of varied intensity on the development of immune organs compared with acute exposure. It is important to evaluate the developmental toxicity induced by long-term exposure to nanoparticles during multiple prenatal periods.

In the present study, dams were exposed to CB-NP on gestational days 8 and 9, which correspond to the period approaching the start of gestational thymus and spleen development. The initial formation of splenic and thymic primordia in mice occur at gestational day 9.5 (Hollander et al., 2006) and 11.5 (Gordon and Manley, 2011), respectively. No significant effects were observed in the pre-implantation period and the fetal developmental period exposure groups, suggesting that nanoparticle exposure during the period of initial formation of lymphoid primordia may trigger the disturbance of fetal immune development and alteration in lymphocyte population in the infants. With the formation of primordia, progenitor cell-derived hematopoietic stem cells migrate and enter the thymic and splenic primordium (Hollander et al., 2006; Hörnblad et al., 2011). Subsequently, thymic progenitor cells interact with stromal microenvironments for T-cell development (Petrie, 2003; Takahama, 2006). On gestational days 14–16 of mice, blood vessels begin to sprout into the thymic primordium, which then allows hematopoietic progenitor cells to access the thymus through the vasculature (Blackburn and Manley, 2004; Gameiro et al., 2010). Dramatic changes in the lymphocyte population in the thymus and spleen of the infant induced by CB-NP exposure on gestational days 9 and 15, as previously shown (El-Sayed et al., 2015) (Figure 7), may be associated with angiogenesis in the thymic primordium. The disturbance in the initial formation of the primordia seems to be exacerbated by additional stimulation of nanoparticles after formation of blood vessels. Repetitive stimuli during the critical developmental stage of lymphoid tissues may cause serious health problems, even at low doses. Furthermore, stimulation during the postnatal period as well as the fetal developmental period may exacerbate the disturbance in the development of the immune system of newborns because the spleen and thymus continue to mature until approximately 21 days after birth in mice (equivalent to 6–8 years of age in humans) (Dietert and Holsapple, 2007). Since humans are usually exposed to air pollutants during both prenatal and postnatal periods, it is necessary to evaluate the combined effects of nanoparticle exposure during the organogenesis and perinatal period to understand developmental immunotoxicity by particulate air pollution, which leads to an increase in the risk of allergic diseases.

The clinical implications of the findings related to changes in the lymphocyte population and their cell types may help in predicting and preventing the diseases related to abnormal development of the immune system owing to the maternal exposure to nanoparticles. CD3 can activate cytotoxic T cells and T helper cells and is primarily used as a T lymphocyte marker. In contrast, B220, also known as CD45R, is mainly expressed on B cells at all developmental stages including Pro-B cell, Pre-B cell, and up to mature B cells, but its expression also observed on NK cell and T cell subsets. Thus, CD3 and B220 generally used in combination for the identification of T cell and B cell. The CD4 and CD8 are frequently used as markers of T helper cells with the surface marker CD4 and cytotoxic T cells with the surface marker CD8. CD4^+^/CD8^+^ phenotype in the thymus mainly includes immature T cells in the developmental stage. It should be noted that CD4^+^, CD8^+^, or double-positive cells include several subsets such as regulatory T cells. The increase in the number of splenic lymphocytes due to CB-NP exposure during organogenesis was observed particularly in the CD3^−^B220^−^ cell number in the lymphocyte subpopulation. Besides, the present study observed the tendency of increase in the CD4^−^CD8^−^ cell number after CB-NP exposure during the organogenesis period. Since CD4^−^CD8^−^ lymphocytes are included in the CD3^−^B220^−^ subpopulation, the alterations in the same cell population likely to be captured in both populations. In fact, our analysis has shown that the increase in the CD4^−^CD8^−^ subpopulation was caused by the increase in the CD3^−^B220^−^ lymphocytes, but not CD3^+^ (T cell) or B220^+^ lymphocytes (B cell). The CD3^−^B220^−^ phenotype in the lymphocyte subpopulation is generally referred to as non-T/non-B lymphocytes, which mainly include innate lymphocytes and mast cells (Yudanin et al., 2019). The cells in the CD3^−^B220^−^ phenotype are important for the innate immune system (Buonocore et al., 2010; Spits et al., 2013), initiation of allergic and/or inflammatory responses via production of key cytokines (von Freeden-Jeffry et al., 1998; Klein Wolterink and Hendriks, 2013; Walker et al., 2013), and contribute to the activation of the adaptive immune system (Galli et al., 2005; Klose and Artis, 2020). The increase in the CD3^−^B220^−^ subpopulation following maternal CB-NP exposure may reflect the induction of inflammation in the fetus and infants. In particular, excessive proliferation of the innate lymphocytes has been observed in patients with asthma (Bartemes et al., 2014; Dunican and Fahy, 2015; Fahy, 2015), atopic dermatitis (Salimi et al., 2013), and chronic rhinosinusitis (Miljkovic et al., 2014). Even though we still failed to acquire further characteristic information for the specific cell type, the increase in the CD3^−^B220^−^ subpopulation by maternal CB-NP exposure may suggest the impairment of the lymphocyte regulation as a potential mechanism underlying developmental immunotoxicity of particulate air pollution containing carbon soots (Fedulov et al., 2008; Latzin et al., 2009). Further investigations are needed to clarify the relationship between the increases in the prevalence of pediatric allergic diseases and disturbance of the CD3^−^B220^−^ lymphocyte population induced by CB-NP exposure during the organogenesis period. Also, since type 2 inflammation contributes primarily to the progression and exacerbation of allergic diseases such as asthma (Brown et al., 2008; Koyasu and Moro, 2011; Licona-Limón et al., 2013; Halim, 2016), it is necessary to analyze the cytokines related to type 2 inflammation. Moreover, the present study cannot reveal the principal cause of the increase in CD3^−^B220^−^ phenotype induced by CB-NP exposure. In particular, the present study evaluated expression levels of genes associated with chemotaxis and master regulator of immune cell differentiation, including *Gata3*, which is an important transcription factor of innate lymphocytes, in the spleen as one of the molecular mechanisms, but no significant differences were observed among groups. Thus, we speculate that it is important to analyze the post-transcriptional regulation such as suppressive microRNA expression for understanding their molecular mechanisms (Dinh et al., 2014; Melo et al., 2019; Bolandi et al., 2020). For example, the previous study reported that overexpression of miRNA-135a results in a significant decrease in the expression level of Gata3 protein, even though only minor changes in the *Gata3* gene expression is observed (Wei et al., 2019). Also, miRNA-27 and miRNA-128 indirectly regulate stabilities of Gata3 protein after transcription and affect lymphocyte differentiation (Guerau-de-Arellano et al., 2011). The evidence suggests that alteration of microRNA expression may be related to the molecular mechanisms underlying the abnormal lymphocyte population induced by CB-NP exposure during the organogenesis period.

Finally, asthma-like symptoms during childhood adversely affect the maturation of lung function leading to chronic obstructive pulmonary disease later in life (Bisgaard et al., 2021). Hence, prevention of asthma caused by particulate air pollution is a challenging issue that should be solved for health promotion of the society as a whole, including children. For realization of the health promotion, it is essential to understand the relationship between particulate air pollution and allergic diseases.

## Conclusion

The present study is the first to evaluate the differential effects of nanoparticles on developmental immunotoxicity with respect to the gestational period of exposure. The organogenesis period, in which the lymphoid primordium formation is initiated, was observed to be the most critical period concerning CB-NP exposure. While no effects were observed after the exposure during pre-implantation and fetal developmental gestational periods, exposure to low doses of CB-NP on gestational days 8 and 9 during the organogenesis period in mice disturbed the lymphocyte population in offspring. Moreover, the CD3^−^B220^−^ phenotype (non-T/non-B lymphocytes), which likely to be involved in innate immune system associated with the pathogenesis of allergic diseases, increased in the organogenesis exposure group. Collectively, the present study revealed the effects of maternal exposure to CB-NP on the development of the thymus and spleen during each stage of gestation. Our findings indicate the importance of focusing on the organogenesis period for evaluation and management of developmental immunotoxicity caused by nanoparticle exposure. Based on the findings of the present and previous research, we can propose that evaluation of combined effects during the organogenesis and perinatal periods are needed to prevent developmental immunotoxicity and to predict the risk of allergic diseases caused by particulate air pollution.

## Data Availability

The raw data supporting the conclusion of this article will be made available by the authors, without undue reservation.

## Abbreviations

ANOVA, analysis of variance; CB-NP, carbon black nanoparticles; FACS, fluorescence-activated cell sorting; H&E, hematoxylin and eosin; IL, interleukin; PND, postnatal day; qRT-PCR, quantitative reverse transcription-polymerase chain reaction; SD, standard deviation; Th2, T helper 2.
